# The Linkage Between Autism Spectrum Disorder and Dup15q Syndrome: A Case Report

**DOI:** 10.7759/cureus.24205

**Published:** 2022-04-17

**Authors:** Erisa Shehi, Herisha Shah, Adityabikram Singh, Vijay S Pampana, Gurjinder Kaur

**Affiliations:** 1 Basic Biomedical Sciences, Touro College of Osteopathic Medicine, Middletown, USA; 2 Basic Biomedical Sciences, Rutgers University New Jersey Medical School, Newark, USA; 3 Neurology, Middletown Medical, Middletown, USA

**Keywords:** pediatrics, developmental and behavioral delay, genetic testing, autism spectrum disorder, angelman syndrome, early intervention

## Abstract

Patients diagnosed with autism spectrum disorder (ASD) frequently have a variable presentation and can suffer from underlying conditions, such as Chromosome 15 abnormalities^]^ The broad diagnosis of ASD and its debilitating symptoms can overshadow underlying conditions and delay crucial interventions. This report describes a male child who was diagnosed with ASD at the early age of 19 months. Hallmark symptoms seen in this case included lack of social eye contact, lack of joint attention, hand-flapping, and missed motor milestones. Genetic methylation assay revealed a duplication on maternally derived chromosome 15, indicating concurrent 15q11-q13 duplication syndrome (Dup15q). Screening assessments for ASD are an important step in the initial management of developmental abnormalities. However, early genetic screening can lead to a more accurate diagnosis, personalized treatment, and better quality of life in patients with atypical symptoms caused by undiagnosed comorbid conditions.

## Introduction

Patients suffering from autism spectrum disorder (ASD) can present with a plethora of coexisting conditions, such as Chromosome 15 abnormalities which include Prader Willi Syndrome (PWS), and Angelman Syndrome (AS), and the focus of this case report, 15q11-q13 duplication syndrome (Dup15q) [1,2]. The Prader-Willi/Angelman Critical Region (PWACR; Chromosome 15q11-13) is considered a potential locus for genes conferring susceptibility to neurodevelopmental issues and ASD [1]. This region has five breakpoints (BP) with local DNA repeats that are often implicated in duplication and deletions. Deletions in the region frequently involve BP1 and BP2/BP3 while duplications are less straightforward, often involving BP4 and BP5 [3]. While PWS and AS results from paternal and maternal microdeletions of the 15q11.2-q13 region respectively, Dup15q is caused by a duplication of the same genetic material.

PWS is characterized by hypotonia, short stature, failure to thrive, behavioral problems, hyperphagia, hypogonadism, and obesity. AS is characterized by symptoms such as paroxysms of laughter, hand-flapping, developmental delays, microcephaly, seizures, ataxia, hypotonia, motor delay, significant intellectual deficit, and severe speech impairment [4]. Dup15q is unique in that it can present characteristics of both PWs and AS as well as some distinct traits. Common among the three disorders features include hypotonia, speech/language disorder, developmental delay, behavioral challenges, and abnormal EEG. These diagnoses are established in a proband who meets the consensus clinical diagnostic criteria and/or presents with molecular genetic testing suggestive of aberrant expression of the PWACR [1,5].

This case report describes a male child who was diagnosed with ASD at an early age of 19 months, in contrast to most diagnoses which usually occur after the third year of life [6]. The current case study is distinctive as it characterizes the early detection of ASD and then concurrent chromosome 15 anomalies such as Dup15q. Although very challenging, this case both reevaluates the high comorbidity between Dup15q and ASD and delineates the profound intellectual disability stemming seen in ASD from the multi-system developmental delay caused by Dup15q and related syndromes. The case highlights the importance of swift genetic testing following an early clinical diagnosis of ASD, to further screen for linked disorders such as Dup15q. This allows effective, personalized, and comprehensive interventions for these patients with comorbid disorders. This tailored treatment, specifically aimed at intellectual limitations and severe speech impairment, can be instrumental in providing an improved standard of living for such patients [7].

## Case presentation

This male patient, presently 6.5 years old, was born prematurely at 27 weeks with a postnatal period that was complicated by intraventricular hemorrhage and sepsis. When the physician first saw the patient at 7-8 months of age, there was evidence of some developmental delay due to the inability to roll over, presence of hypotonia, and frequent infantile spasms. At 11 months, the patient again presented with seizures and a head circumference in the second percentile. The seizures were treated with 200 mg of levetiracetam twice daily, which resolved his condition within seven weeks. This successful treatment resulted in marked “interval improvement in the frequent bilateral temporo-occipital epileptiform discharges seen” on EEG.

At 19 months old the patient was examined for signs and symptoms of developmental delay and diagnosed with ASD. The diagnosis of ASD was made at the Middletown Medical Inc. facility at the age of 19 months; this decision was guided by clinical judgment, the Diagnostic and Statistical Manual of Mental Disorders (DSM-V) criteria, and the Modified Checklist for Autism and Toddlers (M-CHAT) screening questionnaire. ASD was suspected due to classical findings such as a lack of interest in interacting with others and a limited desire to play with toys. The clinician was ultimately guided to this diagnosis due to further deficits in eye contact, joint attention, range of facial expression as well as proto-imperative and declarative pointing.

The patient scored a 9 out of 23 on the M-CHAT questionnaire at his two-year pediatric check-up; this testing is normally done between 16 and 30 months of age and scores above 7 indicate a high risk for ASD or developmental delay. Following his diagnosis, the patient continued to miss multiple developmental milestones. Compared to other children of his age, the patient was mostly nonverbal, and his vocabulary was limited to a few single words. He also persistently exhibited echolalia without any improvement after undergoing speech therapies. Motor symptoms in this patient included hypotonia in all limbs and trunk with significant delays in walking. Most children begin walking at about 12 months, but this patient did not start walking until 33 months and required the use of foot orthotics for support with ambulation.

**Figure 1:**
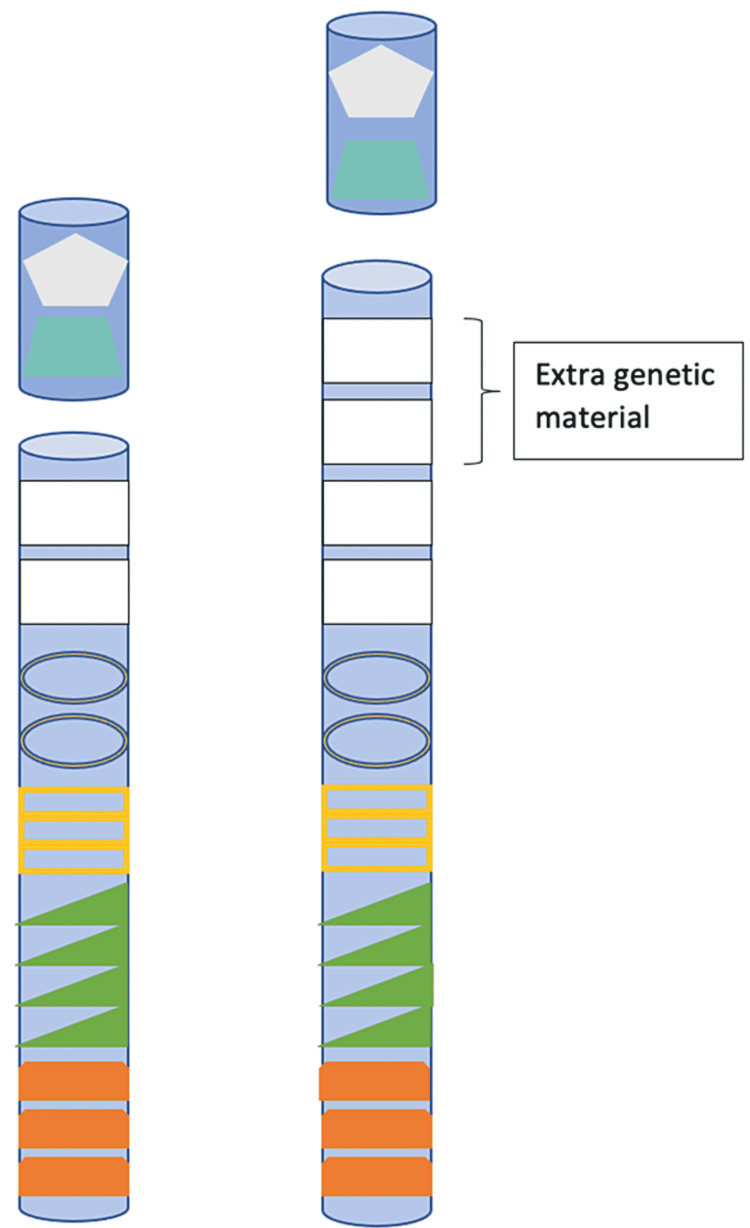
Chromosome 15 alterations in Dup15q syndrome Chromosome 15 with an indication of the duplication of region q11–q13 (white rectangle), as seen in Dup15q Syndrome. The various shapes and colors demonstrate alleles in other regions of the chromosome.

The patient’s significant developmental delay, severe intellectual disability, and speech acquisition difficulties, as well as new symptoms of severe ataxia, hand flapping, and frequent smiling, warranted further testing. Genetic testing revealed Dup15Q with a “pathogenic variant arr(GRch37)15q11.2q13.3, amplification type. A methylation analysis confirmed that copy number gain was on chromosome 15 within the PWACR (Figure 1). Amplification of this region causes a variable phenotype that generally includes central hypotonia, developmental delay, speech difficulties, seizures, behavior disturbances, ASD, and variable degrees of intellectual deficiencies.

The patient’s parent provided written informed consent for the publication of this case report.

## Discussion

There is a challenge in diagnosing ASD as currently there is no one definitive etiology for the disorder. Furthermore, since early intervention is associated with a better outcome, there is greater pressure to find more reliable diagnostic tools [8]. Among the different screening modalities that are available for children with observed developmental delay, one of the most widely accessible and used is M-CHAT at 18 months with a corresponding follow-up M-CHAT at 24 months [6]. Following a clinical diagnosis of ASD, genetic testing is considered the next step as studies show that 10 percent of patients with ASD have a genomic copy number variant, especially in regions of chromosomes 15 and 22 [9]. More specifically in these cases, the alteration of genes in the 15q11-q13 region is associated with PWS, AS, and Dup15q [3,7]. It is important to consider that ASD may be over-diagnosed as both ASD and various chromosome 15 abnormalities have overlapping presentations at early ages [7]. One of the factors that limit the diagnosis of other comorbid genetic diseases is the lack of access to genetic testing [7]. The diagnosis of ASD can be made clinically whereas a diagnosis of Dup15q requires costly genetic screening. Without early and appropriate intervention in children diagnosed with neurodevelopmental disorders, disabilities can be both severe and lifelong in nature with a high burden on families and society alike.

In this patient, the diagnosis of ASD was made through clinical judgment as the patient displayed numerous DSM-V criteria for ASD [10,11]. Additional screening tests such as M-CHAT confirmed the ASD diagnosis [4]. However, the patient showed fewer repetitive and stereotyped behaviors as compared to other children of the same age with ASD. Unique clinical features such as epileptiform activity, behavioral disturbances, and severe developmental delay pointed towards the presence of a comorbid disorder. Genetic methylation screens revealed a copy number gain on maternally derived chromosome 15 (15q.11.2q13.3 microduplication region extending from BP1-5) and this testing was both instrumental and objective in confirming the diagnosis of Dup15q. One could contend if chromosome 15 abnormalities are not identified in such cases, the symptoms at the patient’s initial presentation may lead to over-diagnosis of ASD. Another essential tool for accurate diagnosis is parental genetic testing. The lack of these results in our case is a limitation of this study [12]. Such testing would help elucidate any history of similar disorders in this patient’s family and establish the specific cause of the disease, the parent of origin. Studies have shown that the maternal origin of the duplication is associated with a high risk of recurrence in subsequent children [3]. This determination of recurrence risk through genetic testing is an invaluable tool in family planning and genetic counseling. The MRI is another recommended screening tool for ruling out other associated conditions [13]. Although the patient did not have one done, his diagnosis was already established and the lack of MRI was not a hindrance to his treatment or this study.

Currently, the most widely used treatments for children with ASD are educational and psychological, with some pharmacological treatments being used with moderate benefits at best [14]. Similar treatments such as speech, behavioral and physical therapy are the mainstay of treatment for patients with Dup15q and significantly lessen the burden of the disease. Other treatments directed at symptoms, such as epilepsy, scoliosis, and ambulation difficulties have all been shown to improve outcomes. Hypotonia of the oro-facial musculature can result in feeding difficulties and nutritional deficiencies in patients and must also be addressed [3]. The defining symptoms of Dup15q need to be of special consideration, as targeted therapy can result in significant improvements in quality of life for patients, even more so when diagnosis occurs during a crucial developmental period such as in our case [5]. Future gene therapies which focus on reducing the expression of UBE3A hold promise for patients but are still in development [3].

While the diagnosis of ASD is sufficient in alerting providers to the needs of their patients, patients with rare and undiagnosed genetic abnormalities can have a host of symptoms that may not fit the typical picture of ASD. The findings of our report underscore the importance of genetic testing in patients diagnosed with ASD. It is important that providers maintain a low threshold for genetic testing as the results of these screenings can uncover underlying conditions, guide development of an appropriate treatment plan, and improve the quality of life in patients with ASD and underlying chromosomal abnormalities. This report sheds light on a rare presentation of Dup15q, highlights its concurrence with ASD, and hopes to alert providers on the need for further investigation in ASD patients with atypical presentations.

## Conclusions

This case report details the occurrence of Dup15q in a young child and analyzes its comorbidity with ASD. The strengths of the study included the early use of multiple diagnostic modalities in the identification of developmental disorders in the patient. This report highlights the importance of prompt evaluation and diagnostic testing in any patient with developmental delay. Personalized therapies targeted at speech impairment, motor delay, and language acquisition can significantly improve the quality of life for patients with Dup15q but are only accessible once the proper diagnosis is made. We hope this report can guide providers in initiating timely and comprehensive screening for patients presenting with these atypical symptoms. We also hope to guide further research in the identification of comorbid conditions within ASD and raise awareness for patients with Dup15q and their families.

## References

[1] Baker EK, Godler DE, Bui M (2018). Exploring autism symptoms in an Australian cohort of patients with Prader-Willi and Angelman syndromes. J Neurodev Disord.

[2] Ornoy A, Weinstein-Fudim L, Ergaz Z (2016). Genetic syndromes, maternal diseases and antenatal factors associated with autism spectrum disorders (ASD). Front Neurosci.

[3] Kalsner L, Chamberlain SJ (2015). Prader-Willi, Angelman, and 15q11-q13 duplication syndromes. Pediatr Clin North Am.

[4] Buiting K, Williams C, Horsthemke B (2016). Angelman syndrome - insights into a rare neurogenetic disorder. Nat Rev Neurol.

[5] Dagli AI, Mathews J, Williams CA (1993). Angelman syndrome. https://pubmed.ncbi.nlm.nih.gov/20301323/.

[6] Zwaigenbaum L, Maguire J (2019). Autism screening: Where do we go from here?. Pediatrics.

[7] Trillingsgaard A, ØStergaard JR (2004). Autism in Angelman syndrome: an exploration of comorbidity. Autism.

[8] Nahmias AS, Pellecchia M, Stahmer AC, Mandell DS (2019). Effectiveness of community-based early intervention for children with autism spectrum disorder: a meta-analysis. J Child Psychol Psychiatry.

[9] Griesi-Oliveira K, Sertié AL (2017). Autism spectrum disorders: an updated guide for genetic counseling. Einstein (Sao Paulo).

[10] Thabtah F, Peebles D (2019). Early autism screening: a comprehensive review. Int J Environ Res Public Health.

[11] Hodges H, Fealko C, Soares N (2020). Autism spectrum disorder: definition, epidemiology, causes, and clinical evaluation. Transl Pediatr.

[12] Srivastava S, Love-Nichols JA, Dies KA (2020). Correction: Meta-analysis and multidisciplinary consensus statement: exome sequencing is a first-tier clinical diagnostic test for individuals with neurodevelopmental disorders. Genet Med.

[13] Harting I, Seitz A, Rating D (2009). Abnormal myelination in Angelman syndrome. Eur J Paediatr Neurol.

[14] Zhou MS, Nasir M, Farhat LC, Kook M, Artukoglu BB, Bloch MH (2021). Meta-analysis: pharmacologic treatment of restricted and repetitive behaviors in autism spectrum disorders. J Am Acad Child Adolesc Psychiatry.

